# Attention-deficit hyperactivity disorder (ADHD) and glial integrity: an exploration of associations of cytokines and kynurenine metabolites with symptoms and attention

**DOI:** 10.1186/1744-9081-6-32

**Published:** 2010-06-09

**Authors:** Robert D Oades, Aye-Mu Myint, Maria R Dauvermann, Benno G Schimmelmann, Markus J Schwarz

**Affiliations:** 1 Clinic for Child and Adolescent Psychiatry and Psychotherapy, University of Duisburg-Essen, 45147 Essen, Germany; 2 Laboratory for Psychoneuroimmunology, Ludwig Maximillian's University Psychiatric Hospital, 8036 Munich 2, Germany; 3 Child and Adolescent Psychiatry, University of Bern, Effingerstr. 12, 3011 Bern, Switzerland

## Abstract

**Background:**

In contrast to studies of depression and psychosis, the first part of this study showed no major differences in serum levels of cytokines and tryptophan metabolites between healthy children and those with attention-deficit/hyperactivity disorder of the combined type (ADHD). Yet, small decreases of potentially toxic kynurenine metabolites and increases of cytokines were evident in subgroups. Therefore we examined predictions of biochemical associations with the major symptom clusters, measures of attention and response variability.

**Methods:**

We explored systematically associations of 8 cytokines (indicators of pro/anti-inflammatory function) and 5 tryptophan metabolites with symptom ratings (e.g. anxiety, opposition, inattention) and continuous performance test (CPT) measures (e.g. movement, response time (RT), variability) in 35 ADHD (14 on medication) and 21 control children. Predictions from linear regressions (controlled by the false discovery rate) confirmed or disconfirmed partial correlations accounting for age, body mass and socio-economic status.

**Results:**

**(1) **Total symptom ratings were associated with increases of the interleukins IL-16 and IL-13, where relations of IL-16 (along with decreased S100B) with hyperactivity, and IL-13 with inattention were notable. Opposition ratings were predicted by increased IL-2 in ADHD and IL-6 in control children. **(2) **In the CPT, IL-16 related to motor measures and errors of commission, while IL-13 was associated with errors of omission. Increased RT variability related to lower TNF-α, but to higher IFN-γ levels. **(3) **Tryptophan metabolites were not significantly related to symptoms. But increased tryptophan predicted errors of omission, its breakdown predicted errors of commission and kynurenine levels related to faster RTs.

**Conclusions:**

Many associations were found across diagnostic groups even though they were more marked in one group. This confirms the quantitative trait nature of these features. Conceptually the relationships of the pro- and antiinflammatory cytokines distinguished between behaviours associated more with cognitive or more with motor control respectively. Further study should extend the number of immunological and metabolic markers to confirm or refute the trends reported here and examine their stability from childhood to adolescence in a longitudinal design.

## Background

Essential components of the diagnosis of attention-deficit/hyperactivity disorder of the combined type (ADHD) are clinical impairments of attention, impulsivity and hyperactivity.

Further, the variability of the expression of these symptoms and of neuropsychological measures in the laboratory is viewed as central to the syndrome and has been proposed as an endophenotype of the disorder [1-4]. Neuronal firing must be maintained to sustain a behavioural response. A recent hypothesis [5] proposed that an inefficient supply of energy from glial cells (the lactate shuttle: [6]) might underlie the intra-individual variability in behaviour and the underlying neuronal activity of children with ADHD.

The cytokine and growth factor S100B is a potential marker of glial function. Serum levels arise largely but not exclusively from astrocytes [7]. Increases have been associated with brain damage, major depression, psychosis and dementia [8-10]. However, our pilot study of children with ADHD found that levels of S100B were not unusual, although a tendency for levels to decrease in those with internalizing symptoms was noted [11].

Tryptophan metabolism along the kynurenine pathway takes place primarily in glia [12]. Our study [11] also recorded levels of these metabolites and some cytokines that influence the metabolism [13] as further potential indicators of glial integrity. Relevant to our hypothesis on glial function is that metabolites of kynurenine can be neuroprotective (e.g. kynurenate) or potentially toxic (3-hydroxy-kynurenine, 3-HK) and that the balance between these metabolic routes is modulated by the relative activity of the pro- and anti-inflammatory cytokines [13].

**Figure 1:**
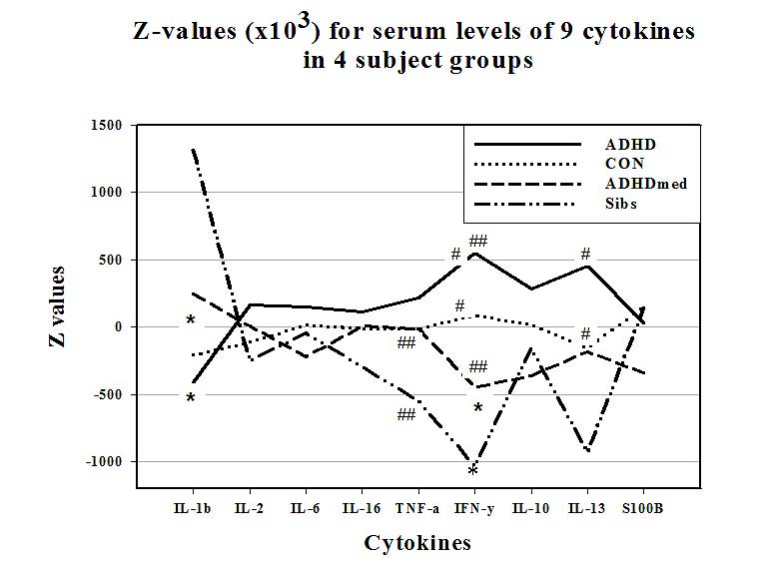
**Shows serum levels (z-values) in 4 groups of subjects for 8 cytokines (the interleukins -1β, -2, -6, -16, TNF-α, IFN-γ, -10, -13)**.  Both control groups tended to have lower IFN-γ levels than the ADHD groups, and the ADHDmed group more IL-1β than the group with ADHD alone. * p = 0.03, # p = 0.1, ## p = 0.07.

Our initial analysis [11] reported a modest imbalance of cytokines in children with ADHD (figure 1). This improved in those treated with methylphenidate, and thus arguably reflected an increased allostatic load [14]. In the kynurenine pathway, we noted lower levels of the potentially toxic 3HK in children with ADHD than controls that could reflect a maturational delay [15] of the neuronal pruning processes seen in typically developing children [16,17]. [However, an alternative metabolic route for kynurenine over nicotine adenine dinucleotide (NAD(+)) rather than the toxic quinolinic acid cannot be excluded [18].]

As we had evidence for differences of the cytokine levels and their associations specific to sub-groups (see internalizing symptoms and S100B above, and interleukin IL-10 and IL-16 in those with experience of allergy), we proposed that there would be associations with the major symptom clusters and underlying processes. In particular, we focused on ratings of inattention, hyperactivity, opposition and anxiety, and laboratory measures of attention where the coefficient of variation as a measure of variability could be calculated. This aim has aetiological significance firstly as the inattentive symptoms are those most likely to persist into adulthood [19], and secondly the measure of variability relates directly to the glial hypothesis for the generation of features of ADHD [5]. On the basis of allostasis we predicted that poorer measures of attention would relate to increased levels of some cytokines and reflect an imbalance between pro-and anti-inflammatory activity.

The absence of previous work in this field makes precise predictions impractical. We know of only one study of cytokines in ADHD [20] and one relating to kynurenine [21]. The former found no extreme values for type-1 or type-2 interleukins among patients with ADHD, unlike those with schizophrenia or obsessive-compulsive disorder. The latter described a potential increase of kynurenine metabolism (over that for serotonin, 5-HT) in ADHD following stimulant medication. Indeed, there is substantial indirect evidence for considering a role for 5-HT in ADHD, and by implication the availability of its precursor tryptophan [22,23]. Thus, in the domain of impulsivity, there is evidence for the genetic control in ADHD of the expression of 5-HT receptors, its synthesis and availability [24]. In the domain of sustained attention, task performance related negatively to 5-HT metabolism, and increased 5-HT metabolism (vs. dopamine metabolism) was inversely related with the signal detection measure, d-prime [25]. In support of a continued consideration of 5-HT metabolism here, we reported moderately increased tryptophan availability in untreated ADHD children [11].

Thus, this study represents an exploration of the hypothesis that there are potentially functional associations of symptom ratings (e.g. inattention) and continuous performance task measures (CPT: sustained attention, impulsivity and variability) with 8 cytokines, tryptophan and 4 tryptophan metabolites. The cytokines include the interleukins IL-2, IL-6, interferon-gamma (IFN-γ), tumour necrosis factor-alpha (TNF-α), IL-16, Il-10, IL-13 and S100B. The metabolites include 5-hydroxy-indoleacetic acid (5-HIAA), kynurenine, kynurenate, and 3HK.

## Methods

### Subjects

Twenty one medication-naïve, consecutively referred children were diagnosed with ADHD combined type (DSM-IV) and recruited to the ADHD-group. Fourteen older children had been given the same diagnosis 4 years earlier and were receiving medication (ADHDmed-group). Their 7 healthy siblings [11] are not further considered here. Controls for the ADHD-group were 21 normally developing children recruited by advertisement. For all groups exclusion criteria included a history of encephalitis, autism, epilepsy, Tourette syndrome, bipolar disorder, IQ ≤ 80, brain damage and any genetic or medical condition associated with externalizing behaviours that might mimic ADHD. The protocol, respecting the Declaration of Helsinki, was approved by the ethics committee of the medical faculty of the University of Duisburg-Essen. Verbal and written information on the study was given to the children and the parents or guardians who gave written consent to the procedures.

Diagnosis was based on a semi-structured interview of the parents (Parental Account of Children's Symptoms, PACS: [26,27]) on mood disorders, ADHD/hyperkinetic syndrome, disruptive behaviour and additional problems of the children in structured and unstructured situations. Following training with written definitions of the behaviours replies were rated for the previous week and year (in a period without medication) on a 4-point scale. A standardized diagnostic algorithm based on the DSM-IV criteria for the symptoms, age of onset, situational pervasiveness and clinical impairment was applied to this information. This procedure has achieved good inter-rater reliability: (r = .76 to .96; [27]). Symptoms were rated with the Conners parent and teacher ratings scales (CPRS-R:L, CTRS-R:L: [28]). Internalizing and externalizing symptoms were recognized on the anxiety and oppositional subscales with T>65 on either the CTRS or CPRS (9/14 [ADHD], 6/2 [controls], and 6/9 [ADHDmed].

Information on gender, age, body-mass index (BMI), IQ, socio-economic index (SES) and reported allergic sensitivities is given in table 1. The IQ was assessed with the CFT-20-R [29] or for children less than 9 years with the Kaufman assessment battery [30]. The current and at-birth reported SES of the father and mother for profession/qualification was scored on a scale of 1-7 [31]. Allergies in the past (> 12 months) and currently experienced were rated for severity (0-3).

**Table 1:** Characteristics of the ADHD, control and ADHDmed groups of children (means and standard deviations)

	*ADHD*		*Control*		*ADHDmed*		
	**mean**	**SD**	**mean**	**SD**	**mean**	**SD**	

**N**	21		21		14		

							

**Gender (m/f)**	14m, 7f		20 m, 1 f		12 m, 2 f		

							

**Age (y)**	8.9**	1.4	11.0**	1.5	12.6	2.1	

							

**IQ**	95.8*	10.7	114.1	14.4	106.7	12.4	

							

**Height (cm)**	132.1	9.3	147.3	11.6	161.2	17.8	

							

**Weight (kg)**	29.2	8.4	39.7	11.7	54.7	19.1	

							

**BMI**	16.5***	2.4	17.9***	3.1	20.5	4.6	

							

**Allergy (N)**	8		11		2		

							

**Allergy (grade)**	0.95	1.2	0.76	0.9	0.29	0.7	

							

**SES-birth (M)**	4.2	1.6	3.6	0.8	3.7	1.4	

**(F)**	4.3	1.6	3.3	2	3.7	1.6	

							

**SES-current (M)**	4.3	2.3	3.3	1.5	4.4	2	

**(F)**	4.3	2.2	3.5	2.1	4.1	1.9	

BMI = Body mass index,

SES = socio-economic scale for the mother (M) and father (F) at child birth and currently (scale 1-7)

Allergy = the number of subjects reporting allergic sensitivity and the rated severity (scale 0-3)

* p < 0.05 ** p < 0.02 *** p < 0.005

### Continuous performance test (CPT/Qb)

The CPT/Qb test provides data on sustained attention: accuracy, response times and their variability and restless movements [32]. It lasts 4-15 minutes depending on the speed of response. A grey circle (target), and another with a cross (nontarget) are presented in a random sequence for 100 ms every 2 sec on a computer monitor. Half of the 450 stimuli require a button-press within 2 sec. Accuracy is measured by the percentage errors of omission (sustained attention) and commission (impulsivity). Multiple and anticipatory responses (< 150 ms) were excluded from the analysis. Response latency measures include the mean reaction time (RT) the variation (standard deviation, SD) and coefficient of variance (intra-individual variability, CV = 100 × RT-SD/RT). During the task the subject's movements are recorded by an infrared camera following a reflective marker on a headband. The camera reads the X/Y coordinates of the marker, sampling 50x/sec and scales (1/27) the measures to mm/sec. The duration of restlessness (time spent active [> 1 cm/sec] as a percentage of task duration) and the number of microevents (movements of >1 mm) are recorded [33].

### Biochemistry

Details are given in the accompanying report [11]. Briefly, fasting venous blood (20 ml) was drawn (08:00-09:00 a.m.), the serum separated and stored at -80°C for analysis blind to the origin of the sample [13]. S100B was measured by an immuno-luminometric assay (detection limit 0.02 μg/L: Elecsys S100™, Roche Diagnostics, Switzerland). The cytokines IL-1β, IL-2, IL-6, IL-10, IL-13, IL-16, IFN-γ and TNF-α were analysed with ELISA test kits from R&D systems. For IL-1β, IL-6, IL-10 and TNF-α high-sensitive assays were used. IL-1β was not detected in 40/63 samples (detection limit: 0.063 pg/ml) and excluded from the correlational analysis.

For amino acid determination the AccQ Tag HPLC method was used (Waters, Milford, MA, USA) and concerned the 5 tryptophan-competing amino acids and 12 others [11]. The fluorescence wave lengths were λex = 250 nm and λem = 395 nm. Analyses for tryptophan and the metabolites were run on a Waters 2695 chromatograph with a 2487 dual-l UV detector and a 2475 fluorescence detector. Tryptophan (lex: 300 nm; lem: 350 nm) and 5-HIAA (lex: 300 nm; lem: 340 nm) were measured with fluorescence, kynurenine (365 nm), kynurenate (330 nm) and 3-HK (365 nm) with UV detection. Three indices of metabolic activity were calculated: (1) T*ryptophan availability *[100 × tryptophan/sum of the competing amino acids], (2) *Tryptophan breakdown *[kynurenine/tryptophan] reflecting the summed metabolic enzymatic activity, and (3a) the *neuroprotective index *[kynurenate/3HK]. Neuroprotection would be increased with larger values. The third index differs from Myint et al. [13] who used the ratio of 1000 × kynurenate/kynurenine (index 3b) representing turnover within the protective pathway rather than the production of toxic metabolites.

### Data analysis

First, group comparisons (Manova) were performed for group characteristics (table 1), symptom ratings and CPT measures, and confirmed with Tukey tests. Symptom subscales for opposition, anxiety, and the DSM scales for inattention, hyperactive-impulsivity and sum scores were analysed on the parent and the teacher scales (table 2). Six CPT responses were selected as representative of motor activity (duration, microevents), attention (errors of omission and commission) and variability (coefficient of variance and RT: table 3). Covariates for gender, age, BMI, SES and IQ were considered and are reported where relevant. Relevance was determined from predictions of group characteristics by the biochemical measures in a backwards step-wise regression. The first results' section summarises these analyses.

**Table 2:** Conners Parent and Teacher Rating Scale Assessments (CPRS/CTRS) of ADHD Symptoms (means and standard errors)

	ADHD	(N = 20) sem	CON	(N = 21) sem	ADHDmed	(N = 14) sem
**CPRS**						
Opposition	71.4	2.1	51.7**	2.1	71.5	2.6
						
Anxiety	56.3	2.3	51.1^#^	2.3	58.2	2.9
						
DSM-IA	72.1	2.2	47.8**	2.2	69.3	2.8
						
DSM-HI	78.4	1.9	48.1**	1.8	72.8	2.3
						
DSM-sum	76.4	1.8	48.0**	1.7	72.6	2.2
						
**CTRS**						
opposition	63.3	2.5	50.8	2.4	56.0	3.1
						
Anxiety	60.3	2.8	54.8	2.6	62.3	3.4
						
DSM-IA	65.8	2.5	48.6*	2.3	59.9	3.0
						
DSM-HI	67.7	2.5	48.4*	2.3	51.5	3.0
						
DSM-sum	68.1	2.3	48.5*	2.2	57.0	2.9

For all subscales (except anxiety) the ADHD groups scored higher than children without a diagnosis (independent of 4 covariates): IA, inattention; HI, hyperactivity-impulsivity: # F(2,49) = 2.7 p = 0.077, η^2 ^= 0.099; * F(2,49) = 7-11, p < 0.002 η^2 ^= 0.23-0.32; ** F(2,49) = 34-74, p < 0.000, η^2 ^= 0.49-0.69;

**Table 3:** Continuous performance task measures (CPT/Qb) of motor activity, response speed/variability and accuracy/errors

CPT/Qb measure	Group	N	Mean	SD	df 2,50
					

**Duration active**	ADHD	21	43.4	23.7	

**(% > 1 cm/sec)**	CON	21	15.6	11	F 5.6, p = 0.006, η2 = 0.18

	ADHDmed	13	23.5^2^	21.9	

					

**Microevents**	ADHD	21	10167	5624	

**(N > 1 mm)**	CON	21	4390	1962	F 5.0, p = 0.01, η2 = 0.17

	ADHDmed	13	5531^1^	3626	

					

**RT (ms)**	ADHD	21	581	85	

	CON	21	444	92	F 4.1, p = 0.02, η2 = 0.14

	ADHDmed	13	533	141	

					

**CV**	ADHD	21	44.1	15.4	

**100 × RT-SD/RT**	CON	21	29.2	5.1	F 5.2, p = 0.009, η2 = 0.17

	ADHDmed	13	33^3^	7.7	

					

**Errors**					

**Omissions**	ADHD	21	20.4	17	

	CON	21	2	3	F 5.6, p = .006, η2 = 0.18

	ADHDmed	13	19.4	24.2	

					

**Commissions**	ADHD	21	17.3	9.1	

	CON	21	9.7	7.4	NS

	ADHDmed	13	6.4^4^	9.7	

ADHD vs. ADHDmed: 1. p = 0.006, 2. p = 0.014, 3. p = 0.013, 4. p = 0.002

The second results section reports the associations sought between the group features and the functional domains of attention and symptoms with the 3 sets of biochemical measures (cytokines, tryptophan metabolism and metabolic indices). Initially in this exploration partial correlations were calculated accounting for the relevant covariates (see above) for each group. Second these results were confirmed/disconfirmed with linear regression analysis to test the hypotheses that the biochemical measures predict the symptoms and attentional features. This assists in the elimination of false positives in the correlational analysis. Applying the false discovery rate to the results of the multiple regressions identifies uncorrected probabilities reported as ≤ 0.01 as satisfying α = 5%: values above this threshold are described as trends or tendencies (p < 0.05/α < 10%). Following each analytical section there is a short summary of the confirmed effects. For tryptophan metabolism measures of tryptophan, kynurenine, kynurenate, 3HK and 5-HIAA and separately the metabolic indices (above) were considered. For the cytokine associations analyses of S100B, IL-2, IL-6, IL-16, TNF-α, IFN-γ, IL-10 and IL-13 measures were considered. A few missing values were replaced by the mean.

## Results

### (1) Group comparisons

#### Group features and comparisons of their symptoms and attentive behaviour

The three groups did not differ on gender distribution, socio-economic scale or incidence of allergy (table 1). ADHD children were younger than the controls and both were younger than the ADHDmed group (p < 0.02): they also had a lower BMI than the ADHDmed group (p < 0.005) and their IQ was lower than in either the ADHDmed or control groups (p < 0.05).

Covarying for age and IQ, the CPRS symptom ratings were clearly higher for both ADHD groups than the controls on each scale except anxiety. The anxiety ratings were only a little higher in the ADHD groups than in controls. Similar but less marked differences were shown with the CTRS. There were no differences between the ADHD groups (table 2). Using the same covariates attenuated the group differences on CPT measures, but only that for the errors of commission errors lost significance. Compared to the ADHD groups controls showed less motor activity, a shorter RT with less variability and more errors of omission (table 3). While post-hoc comparisons of the two ADHD groups showed that medication did not significantly reduce RT or errors of omission, the ADHDmed group did show fewer microevents, a shorter duration of activity, less RT variability and fewer errors of commission.

#### Group comparisons: tryptophan metabolism and cytokines

**Figure 2:**
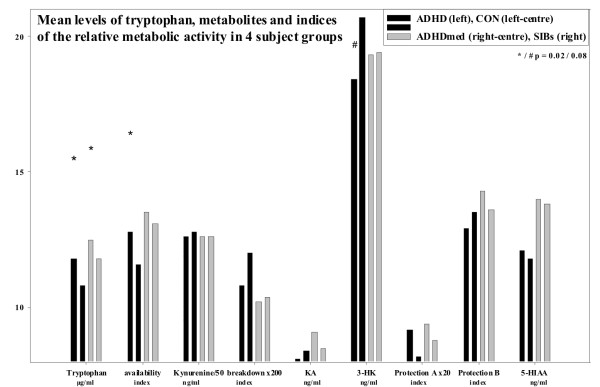
**shows a comparison for the 4 groups of subjects of the levels of tryptophan, its metabolites and the indices for tryptophan availability, breakdown and neuroprotection (A and B)**. Tryptophan levels and availability were higher in both ADHD groups (p < 0.02). Descriptively higher levels of 3HK in the controls (p < 0.08) related closely to Kynurenine levels (r = + 0.85, p < 0.000), but a correlation was absent in the ADHD group (p = 0.35).

Tryptophan levels and availability were modestly higher and breakdown indices lower in those with a diagnosis of ADHD after controlling for age and gender (figure 2, [11]). But this was not reflected in altered levels of 5-HIAA or kynurenine. Taking account of age and BMI, the ratio of kynurenate to 3HK, was lower in children with ADHD than the comparison groups which showed slightly increased levels of 3HK.

There were no group differences in S100B levels, but reductions in ADHD children reflected those with internalizing rather than conduct problems [11]. Except for IL-1β and TNF-α the normalised cytokine values (figure 1) tended to be higher in the ADHD than in the control group. All values in the ADHDmed group returned to more normal levels.

### (2) Relations of biochemical measures to group features, symptoms and attention

#### Group features (relations to tryptophan metabolism and the cytokines)

Across all children tryptophan levels increased with BMI and age (R^2 ^0.14, F(1,54) = 8.69: β +0.37, p = 0.005): this was evident in both the ADHD and ADHDmed groups (R^2 ^0.22/0.60, F(1,19) = 5.33/8.16: β + 0.47/+0.96, p = 0.03/0.002, respectively). 3HK levels also tended to increase in controls (R^2 ^0.20, F(1,19) = 4.69, β +0.45, p = 0.04). IQ was unrelated, but low SES was predicted by lower tryptophan and increasing kynurenate levels (R^2 ^0.15, F(2,53) = 4.70: β + 0.33/-0.34, p = 0.018/0.015, respectively). This was supported by modest associations with increased tryptophan breakdown and lower neuroprotection ratio-B (β -0.3/-0.28, p = 0.02/0.03). Lastly, across all children (and within each group), those with an experience of allergy tended to be predicted by low levels of 5-HIAA (R^2 ^0.07, F(1,54) = 3.93: β -0.26, p = 0.05).

Group features were only marginally predicted by the levels of a few proinflammatory cytokines. Across all subjects increasing TNF-α and decreasing IFN-γ levels tended to predict increasing age (R^2 ^0.084, F(2,53) = 0.09: β +0.28/-0.31, p = 0.077/0.049, respectively). BMI also tended to be predicted by increasing TNF-α and IL-6 levels (β +0.32/+0.45, p = 0.03/0.001, respectively).

Across subjects allergic experience tended to relate to decreasing proinflammatory TNF-α but increasing antiinflammatory IL-10 levels (R^2 ^0.16, F(2,53) = 5.0, p = 0.01: β -0.27/ + 0.31, p = 0.039/0.017, respectively). In the ADHD group, in addition to the TNF-α decrease, (β -0.63, p = 0.03) increasing antiinflammatory IL-13 levels predicted response (β +0.56, p = 0.01). In contrast, for the controls alone the increase in IL-10 was masked by *decreases *of IL-13 in the prediction of allergy severity (R^2 ^0.42, F(2,18) = 6.6, p = 0.007: β + 0.33/-0.13, p = 0.08/0.01, respectively).

#### Summary

Tryptophan levels increased with age and BMI, but lower SES status facilitated metabolism in the kynurenine pathway. Some proinflammatory cytokine levels decreased (IFN-γ) or increased with BMI (TNF-α, IL-6). Independent of diagnosis, children with allergies tended to show lower levels of 5-HT metabolism. Most children with experience of allergy showed decreases of the proinflammatory TNF-α. However, in contrast to the controls who showed decreases of the anti-inflammatory IL-13, the ADHD group showed increased IL-13 levels.

#### Symptom ratings: relations of tryptophan metabolism

In general, symptoms were not associated clearly with tryptophan metabolism. Partial correlations accounting for age, BMI and SES suggested that inattentive symptoms were related to tryptophan levels across all children (r = +0.32, p = 0.026). Alone for controls anxiety related to 5-HIAA levels (r = 0.52, p = 0.038) and inattentive ratings with 3HK (r = +0.49, p = 0.037). Only the latter result (3HK) approached the threshold for significance in the regression (R^2 ^0.19, F(1,19) = 4.5: β +0.44, p = 0.048).

#### Symptom ratings: relations of cytokine measures

Overall, considering total symptom ratings in the ADHD group the best predictors were increasing levels of IL-13 and IL-16 along with decreasing levels of S100B (R^2 ^0.57, F(4,16) = 5.21, p = 0.007: β +0.59, +0.51, -0.54, p = 0.024, 0.019, 0.013, respectively) with a minor contribution from TNF-α.

**Figure 3:**
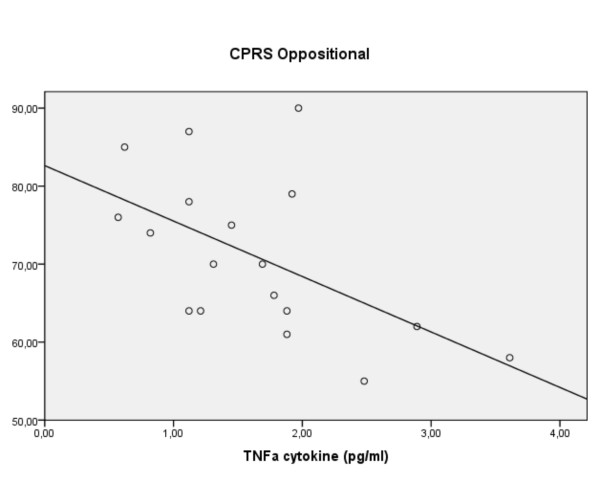
**For children with ADHD, ratings of oppositional behaviour vs. levels of TNF-α (linear regression)**.

**Figure 4:**
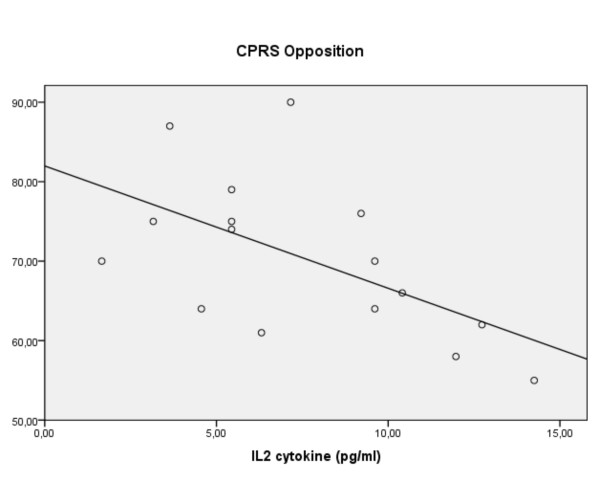
**For children with ADHD, ratings of oppositional behaviour vs. levels of IL-2 (linear regression)**.

Partial correlations accounting for age, BMI and SES pointed to associations for pro-inflammatory cytokines with two symptom groupings, namely the oppositional and hyperactive-impulsive ratings. In the ADHD group decreasing IL-2 (r -0.64, p = 0.026) and TNF-α (r -0.62, p = 0.018: figure 3) correlated with increased ratings of opposition. Regression confirmed the IL-2 result (R^2 ^0.41, F(2,18) = 6.2, p = 0.009: β -0.55, p = 0.007: figure 4) and implied a modest predictive role for increasing levels of S100B (β +0.37 p = 0.057). Of interest is that in the controls another proinflammatory cytokine (IL-6) correlated with oppositional ratings (r +0.48, p = 0.05): the predictive role was confirmed in the regression (R^2 ^0.30, F(1,19) = 7.99, p = 0.01: β +0.54, p = 0.01).

**Figure 5:**
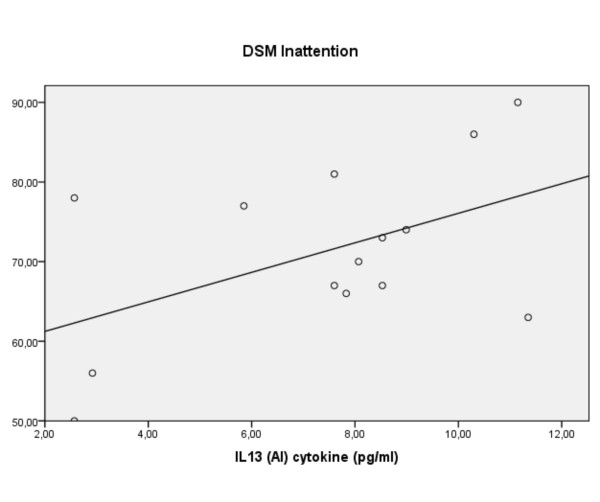
**For children with ADHD, ratings of inattention vs. levels of IL-13 (linear regression)**.

The association of hyperactive-impulsive ratings in the ADHD group with IL-16 (r +0.66, p = 0.015) was confirmed by the regression (R^2 ^0.52, F(2,18) = 9.82, p = 0.001: β +0.80, p = 0.000) with modest support from decreasing levels of S100B (β -0.42, p = 0.035). Anxiety ratings were without relationships to the cytokines. But, intriguingly ratings of inattention in the ADHD group tended to relate positively to levels of the antiinflammatory IL-13, yet negatively to IL-13 in the controls (R^2 ^0.33/0.45, F(2/3, 18/17) = 4.5/4.6, p = 0.026/0.015: β +0.44/-0.67, p = 0.03/0.007, respectively: figure 5). The ADHD result was modestly supported by increasing IL-6 levels (β +0.38) and the control result by increasing S100B (β + 0.64).

#### Summary

There were modest associations of tryptophan levels and its metabolism to the potentially toxic 3HK with inattention, and 5-HT metabolism to anxiety. Proinflammatory cytokines were associated with oppositional symptoms: decreases of IL-2 in the ADHD group contrasted with increases of IL-6 in the controls. In general, ADHD symptoms were associated with increases of the antiinflammatory IL-13 (inattention), increases of IL-16 (hyperactive-impulsive symptoms) and with decreases of S100B (total symptoms).

#### CPT performance: relations of tryptophan metabolism

Across all subjects motor activity (duration, microevents) showed a very modest tendency to increase with lower levels of 3HK (R^2 ^0.07, F(1,54) = 4.0/3.4: β-0.26/-0.24, p = 0.05/0.07, respectively), but this was not evident in the partial correlations and single group analyses.

**Figure 6:**
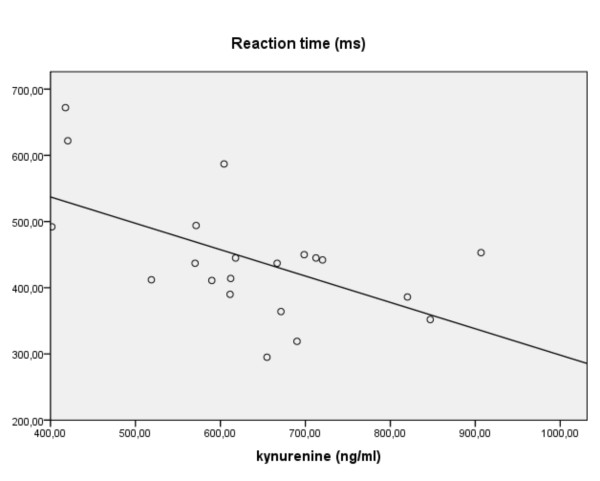
**For healthy control children, reaction time (RT) on the CPT task vs. levels of kynurenine (linear regression)**.

In control children faster RTs were associated with increasing kynurenine levels (R^2 ^0.32, F(1,19) = 9.13: β -0.57, p = 0.007: figure 6). This confirmed the partial correlation (r = -0.52, p = 0.026). Unexpectedly, the opposite relationship appeared in the ADHDmed group (r = + 0.93, p = 0.000: R^2 ^0.48, F(1,12) = 10.93: β +.69, p = 0.006) with no association evident in the non-medicated cases. Across all subjects, there was a partial correlation for the coefficient of RT variance with tryptophan levels (r+0.30, p = 0.035), yet a predictive role for tryptophan was not confirmed in the regression. Interestingly, the decrease of tryptophan (and variability) in controls (vs. the ADHD group, figure 2) tended to be reflected by increases of 5-HIAA (r = -0.45, R^2 ^0.17, F(1,19) = 3.95: β -0.42, p = 0.06). But, no associations were evident in the ADHD groups. The interpretation of the RT associations in terms of 5-HT metabolism is supported by the partial correlations of the turnover indices. While tryptophan availability was positively related to RT (r+0.36, p = 0.012), the breakdown to kynurenine was negatively associated (r-0.31, p = 0.03).

The error analysis suggested that omissions were positively associated with tryptophan levels across subjects (r = +0.38 p = 0.006: R^2 ^0.14, F(1,54) = 9.03: β +0.38, p = 0.004). This positive association between tryptophan levels and omission errors was evident as a trend in the ADHDmed group where the relationship to commission errors was negative (R^2 ^0.35/0.54, F(1,12)/(2,11) = 6.37/6.42: β +0.59/-0.92, p = 0.027/0.004, respectively). This negative trend with commission errors is upheld by their association with the tryptophan breakdown index (R^2 ^0.49, F(1,12) = 11.8: β +0.70, p = 0.005).

#### CPT performance: relations of cytokine measures

Considering motor activity across all subjects, the partial correlations pointed to a positive association of the duration measure with IL-16 levels (r+0.30, p = 0.038). This was confirmed by the regression (R^2 ^0.08, F(1,54) = 4.56: β +0.28, p = 0.037). The motor associations appeared to be driven by IL-16 in the ADHD group for both duration and microevent measures (R^2 ^0.55/0.47, F(3,17) = 6.9/5.0, p = 0.003/0.01: β +0.55/+0.47, p = 0.007/0.028, respectively). [The other degrees of freedom here refer to minor contributions from decreases of the pro-inflammatory IL-6 and TNF-α.] In contrast, the effect of medication introduced a potential modest antiinflammatory influence (e.g. IL-10 with microevents, R^2 ^0.35, F(1,12) = 6.3: β +0.59, p = 0.027).

**Figure 7:**
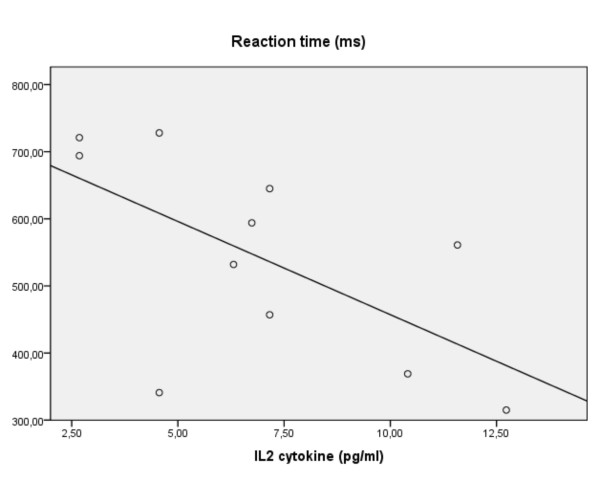
**For children with ADHD on medication the reaction time vs. levels of IL-2 (linear regression)**.

**Figure 8:**
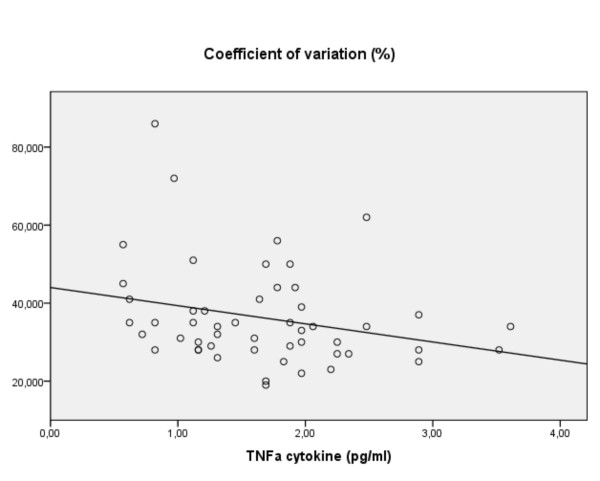
**For all subjects, the coefficient of variance (CV) vs. levels of TNF-α (linear regression)**.

Several analyses indicated minor negative associations of RT with cytokines (e.g. IL-2, S100B) but only the effect of medication showed that decreasing RTs related to increasing levels of IL-2 (ADHDmed: β -0.73, p = 0.003: figure 7). This contrasts with RT variability, where across subjects the coefficient of variance was influenced in opposite ways by TNF-α and IFN-γ (R^2 ^0.18, F(2,53) = 6.1, p = 0.004: β -0.46/+0.04, p = 0.002/0.007, respectively: figure 8). While these two relationships were not driven by processes related to diagnosis, the association with TNF-α was most marked in the medicated ADHD group (β -0.70, p = 0.007).

**Figure 9:**
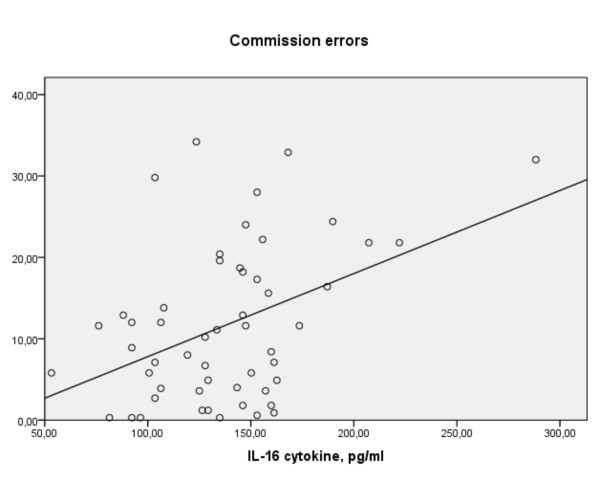
**For all subjects, commission errors on the continuous performance test vs. levels of IL-16 (linear regression)**.

In the error analyses errors of commission were predicted by levels of IL-16 (r+0.42, p = 0.003: R^2 ^0.17, F(1,54) = 10.75: β +0.41, p = 0.002: figure 9). But, as with variability (above) this was most apparent in the ADHDmed group (β +0.75, p = 0.000). In this group alone, again as with variability, decreases of proinflammatory TNF-α (β-0.36, p = 0.016) and IL-6 (β-0.39, p = 0.032) also tended to predict errors of commission. For omission errors only the negative associations of IL-13 (ADHD: β-0.47, p = 0.03)) and IL-16 (ADHDmed: β-0.63, p = 0.004) were of note.

#### Summary

Measures of motor activity were not related to tryptophan metabolism but were positively predicted by IL-16 levels. In contrast, shorter RTs were associated with increasing kynurenine levels in controls, but by decreasing levels in the ADHDmed group. Here, the cytokines were without any major influence. Interestingly, decreased RT variability in controls was reflected by modest increases of catabolism to 5-HIAA. With regard to the cytokines, across subjects, levels of variability reflected the opposing influences of TNF-α and IFN-γ. In the error analysis, while the build up of tryptophan predicted omission errors, its breakdown predicted errors of commission. But only the errors of commission were predicted by IL-16 levels. Medication may moderate interactions of pro-and antiinflammatory cytokines in motor activity (IL-2/RT, IL-10/microevents) and the influences of proinflammatory TNF-α, IL-6 (variable RT) and IL-16 (impulsivity).

## Discussion

Over 20 years ago Kim and Choi [34] demonstrated that the breakdown of tryptophan and kynurenine can produce toxic metabolites (3HK and quinolinate) in neocortical tissue, but an alternative pathway produces kynurenate that can be protective. Kynurenine metabolism in the brain occurs largely in the glia that envelop glutamatergic synapses [12]. Among the numerous responses of the glia to excitotoxicity are alterations in the proinflammatory cytokines, such as TNF-α and IFN-γ [35], where the balance with antiinflammatory cytokines influences tryptophan metabolism [13]. Recently there have been numerous reports of associations of kynurenine metabolites and cytokines with brain damage and psychiatric disorders [8,36-40]. While we only found modest changes of selected markers of these cytokines and metabolites in our pilot study of ADHD [11], we hypothesized here that key symptoms and poor attentional function would reflect alterations of the activity of these markers. Features of the kynurenine metabolic pathway and neuroinflammatory activity of cytokines have not been explored before in the context of ADHD.

We report a preliminary exploration of whether products of tryptophan metabolism and the cytokines that modulate this metabolism predict some of these symptoms, and their laboratory measures in children with a strict diagnosis of ADHD (combined type) contrasted with those developing normally. The study is predicated on increasing evidence that alterations in these two biochemical domains are reflected in both the features of ADHD [21,41] and animal models of these functions [42,43].

Those with the diagnosis of ADHD had clearly more symptoms of inattention, hyperactivity-impulsivity and opposition than those without the diagnosis. On a test of sustained attention the children with ADHD showed more motor activity, errors of omission, a slower RT and more RT variability. Most cytokine levels were modestly increased in children with ADHD (not S100B and IL-1β) and all tended to normalize on psychostimulant medication. While tryptophan availability was slightly increased in the ADHD group, 3HK levels tended to be lower than in the controls [11].

### The cytokines and associations with symptoms

The DSM ratings of total symptom severity in the ADHD group were predicted by increasing levels of IL-16, and the anti-inflammatory IL-13, but by decreasing levels of S100B. The anti-inflammatory relationship was modestly evident for inattentive symptoms in the ADHD group, and arguably effective in countering dysfunction in that higher levels of IL-13 in the controls were related to fewer symptoms of inattention. The role of IL-16 can be pro-or anti-inflammatory in different situations [44,45]. Here, in the ADHD group, IL-16 levels additionally predicted hyperactive-impulsive symptoms that were also modestly associated with decreased S100B. The IL-16 relationship with hyperactive symptoms might not be expected in so far as 2 genetic polymorphisms have been associated with the inattentive type of ADHD [46]. However, the difference may reflect other features of the two populations influencing the neuroinflammatory role of IL-16, such as allergy (see below). The association of symptom severity with decreasing S100B levels is consistent with our prediction of inefficient glial function in ADHD [11] rather than an overproduction resulting from apoptosis and brain damage reported for other major psychiatric disorders [10,47,48].

Only proinflammatory cytokine activity related to oppositional symptom ratings. Decreasing levels of IL-2, and to a minor extent TNF-a, were associated with increasing signs of opposition. We suggest the negative direction of association mirrors consistently the frequent reports of increased IL-2 activity in disorders such as schizophrenia and Parkinsonism with a very different clinical profile [40,49,50]. On the other hand we note a relationship between increasing IL-6 and oppositional behaviour in the control group. We tentatively suggest that, as IL-6 levels increased with BMI and the controls were marginally older than the patients, such an increase reflects the normal parallel development of boisterous behaviour often associated with adolescence onset. This interpretation is also consistent with the well-studied associations of IL-6 levels with physical and psychological stress [51].

Sensitivity to allergy reflected opposing trends for pro-and antiinflammatory cytokines. Children sensitive to allergy showed 15% more IL-16, 32% more IL-13 and similar levels of IL-10 [11]. But for the ADHD children who experienced allergic responses, these responses tended to be predicted by decreasing levels of the proinflammatory TNF-α and increases of the antiinflammatory IL-13. For controls the trend went in the opposite direction for IL-13, but this was balanced by modest increases of another antiinflammatory cytokine, IL-10. The difference between groups in the proinflammatory response is quantitative, but that for the antiinflammatory response is qualitative. This physiological response may underlie a subgroup of ADHD children with high allergic responsivity and for whom ADHD symptoms are alleviated by removal of the allergens from the diet [52-54]. Further, IL-10 secretion is facilitated by physical or psychological stress [55] and could be responsible for the decreases of tryptophan metabolism [56] discussed below.

### The cytokines and associations with CPT performance

During the CPT both the duration of major movements and the quantity of small fidgety activity (microevents) were directly measured. Increasing IL-16 levels not only predicted hyperactive symptoms (above) but clearly predicted quantitative measures of motor activity, especially (but not only) in ADHD children. The associations extended to error omission and commission in the medicated children that may reflect motor as well as cognitive control. The associations of IL-16 with motor activity and allergy in this study clearly add to the concept of this being a pleiotropic cytokine, for which not all the roles have yet been elaborated.

Accuracy in terms of the errors made is more usually thought of as a cognitive than a motoric feature. Our data suggest that decreasing levels of the anti-inflammatory IL-13 may not only relate to inattentive symptoms (above), but specifically to increases of errors of omission. But, both this association and the tendency for errors of commission in medicated children to be reduced as pro-inflammatory TNF-α and IL-6 levels increased were modest trends. This should be examined closely in future studies. Nonetheless the increase of IL-2 with decreased RT in the ADHDmed group supports the proinflammatory effect on attention-related function.

Associations of the coefficient of variance of the RT is of especial interest, first as variability is the core feature of the hypothesis of glial impairment in ADHD [5], and secondly as it has been proposed as an endophenotype for ADHD (introduction). Here, across all subjects, increasing RT variability was related to decreasing levels of TNF-α and with increasing levels of IFN-γ.

Numerous studies confirm the involvement of proinflammatory cytokines in cognitive performance both in animal models and in the treatment of cancer patients. For example IL-2 treatment can interfere with long term potentiation in the hippocampal slice [57] and the formation of working memory in patients [58]. Treating rodents with IL-6 can impair measures of memory [59,60], yet in knockout mice recall can be facilitated [61,62]: the relationship of IL-6 to cognitive function may be linear or U-shaped [63]. However, IL-6 expression depends on the nature of the allele carried (CC/GC genotype) and this variable has too rarely been considered [64,65]: the C allele may dominate in ADHD while the protective G allele may predominate in typically developing children [66]. The characteristics of TNF-α and IFN-γ have much in common with other proinflammatory interleukins. Too much [67] or too little TNF-α [68] can impair cognitive function, but moderate levels are essential [69]. Variation of these levels can influence the turnover of each of the monoamines [70]. Thus it is no surprise that TNF-α levels relate to accuracy and the variability of response in ADHD. However, the relationship of the effects with those of IFN-γ require closer study, as the physiological response to each varies with the measure and over time [70].

### Tryptophan metabolism and associations with symptoms and CPT performance

Even after controlling for age, BMI and SES there were tendencies across subjects for a build-up of tryptophan (and its increased availability) to be associated with symptoms of inattention, RT and its variability as well as increases of errors of omission. In contrast decreased tryptophan levels (more breakdown) related to errors of commission. However, tryptophan metabolism was not related to motor activity. This was unexpected as the motor regions of the brain receive a dense 5-HT innervation [71].

For controls, slower RTs but for the ADHDmed group faster RTs were clearly associated with lower kynurenine levels. There was an indication that low levels of kynurenine for controls was reflected by the trend for reduced variability and an associated increase of metabolism in the 5-HT pathway. For medicated children the implication is of further glial metabolism in the kynurenine pathway that is facilitated by psychostimulant treatment [72]. Other trend relationships (e.g. 3HK with inattention, 5-HIAA with anxiety) were equivocal. The lack of anticipated changes of 5-HT metabolism related to antisocial/oppositional behaviour [73,74] may simply reflect the extent of 5-HT metabolism from peripheral sources.

The absence of clear associations for tryptophan metabolism with symptoms or attention related performance in ADHD is itself of interest. An overexpression of indoleamine 2, 3 dioxygenase facilitates degradation to kynurenine (and its potentially toxic metabolites) and reduced 5-HT metabolism [75]. Such activity is promoted by proinflammatory cytokines [76]. This scenario fits evidence for the development of psychotic symptoms [38] and major depression [13]. But, our results tend to support the opposite effect in ADHD with increases of tryptophan availability relating to inattention and variable responses. Indeed, here there was even modest support for previous evidence for an involvement of 5-HT metabolism [77].

We had anticipated that the cytokine balance would favour kynurenine breakdown to either kynurenate or 3HK. On the basis of their glutamatergic antagonist and agonist activity these metabolites (and the 3HK metabolite quinolinate) have been ascribed a neuroprotective and toxic role, respectively [78]. But in fact our typically developing children rather than the ADHD group showed descriptively higher levels of kynurenate and 3HK. Yet, these measures did not predict symptoms or CPT performance. We speculate that the ADHD children in fact exhibit a relative lack of some of the other characteristics of these metabolites. For example, kynurenate also has α7 cholinergic antagonist properties [79] and higher levels antagonize rather than facilitate AMPA receptor transmission [80]. 3HK has antioxidant capability ([81]) as well as the toxic potential to assist in developmental pruning [16]. Indeed, it is unclear as yet whether energy metabolism in developing children would be impaired by the NMDA agonism of quinolinate [82] or facilitated through further metabolism to NAD(+) [83].

### Limitations

Our conclusions are severely curtailed by not having measured other metabolites (picolinic and, quinolinic acid, NAD(+)), enzyme activity or the expression of genetic variants for these. Also in a pilot study the robustness of the data is naturally compromised by the small number of children that could be recruited and persuaded to offer a blood sample. However, this exploration with 56 children indicates a number of lines of inquiry that are not worth pursuing and others that might merit further study. Indeed, although the number of variables examined raises questions about the prevalence of false positive results, the statistical procedure included confirmatory tests and false discovery assessment to limit this. Cytokine measures, renowned for their variability, were relatively consistent with remarkably few outliers. Future work should seek replication with a longitudinal design to demonstrate consistency of the data in a larger sample. Such a design should account for developmental age, match for gender, and control for medication yet recruit cases with a range of comorbid attributes to clarify their contribution to the diagnostic condition.

## Conclusions

We hypothesised that even if there are no major dichotomies for this selection of metabolites and cytokines between the groups studied, there would be some associations between symptom dimensions or attentional features and the activity of individual markers. In this exploration we have been able to point out some such associations that bear further study. Some regressions predicted 10 to 40% of the variance of the variable in question.

While in most cases the elements of the kynurenine pathway analysed did not show strong associations with behaviour, we are aware that those metabolites determining toxicity or energy supply were missing (e.g. quinolinic acid, NAD(+)). Only such a determination can link unequivocally putative maturation-related pruning (3HK levels) or glial energy supply [5] to metabolism. However, independent of kynurenine metabolism, among the cytokines there appears to be a conceptual division between the activity of antiinflammatory members with motor-control related features (symptoms, microevents) and pro-inflammatory members with cognitive-control features (opposition, variability). In several instances the associations extended across all subjects implying their quantitative trait nature. We recommend replications to include the pro-inflammatory IL-1β and antiinflammatory IL-4 and comparisons to examine stability of these associations before and after adolescence.

## Abbreviations

ADHD: Attention-deficit/hyperactivity disorder; BMI: Body mass index; DSM-IV: Diagnostic and Statistical Manual of the American Psychiatric Association, 4^th ^edition; 5-HIAA: 5-hydroxy-indole-acetic acid; 5-HT: serotonin; HPLC: High Performance Liquid Chromatography; 5-HT: Serotonin; IL: Interleukin; IFN-γ, Interferon gamma; 3HK: 3-hydroxy-kynurenine; PACS: Parental Account of Children's Symptoms; RT: Response time; SES: Socio-economic scale; SD: Standard Deviation; TNF-α: Tumour necrosis factor alpha

## Competing interests

RDO, MD and BGS declare that they have no competing interests beyond the source of finance for the study (see below). AMM & MJS have patented the use of tryptophan pathway metabolites as neurodegenerative markers for depression and related psychiatric diseases

## Authors' contributions

RDO was involved in conceiving and organizing the study as well as analysing the data and writing the report. The organization was refined by BGS who with MD was involved in recruitment, diagnosis and logistics. MJS and AMM advised on the biochemical items for study, organized and ran the biochemical analyses and contributed to the strategy for the study and report. The ideas and hypotheses derived from discussion of the work of AMM and RDO. All authors contributed to and approved the final manuscript.

## Authors' information

The study design was intentionally exploratory and the interpretation accordingly conditional. The presentation is aimed at informing both the disciplines of ADHD (psychiatric) and psychoimmunology (basic research) for whom certain elements may be more or less familiar and include negative and positive findings that may both guide future investigations.

Some of these data were communicated at the 10^th ^Psychoimmunology Expert meeting at Ulm/Günzburg, 12-14^th ^Nov. 2009 [84].
